# Supplementary material for: Aggregation pheromone 4-vinylanisole promotes the synchrony of sexual maturation in female locusts
Source: eLife. 2022 Mar 8;11:e74581. doi: 10.7554/eLife.74581 (PMC8903828; doi:10.7554/eLife.74581)
Supplement: Supplementary file 2. [file elife-74581-supp2.docx]

**Supplementary file 2. Primers used in qPCR analysis.**

| Gene name | Forward primer | Reverse primer |
| --- | --- | --- |
| Vg | CCCACAAGAAGCACAGAACG | TTGGTCGCCATCAACAGAAG |
| JHAMT | AACAACAACAACAAGAGCGGAA | CGTTCGGATCTCCATCGTGTC |
| JHE | GAGTCATTCAACCAGCGCAA | CCAGAACTCCACCACCTCAT |
| LmigOr35 | GTCGGTGGAGCGAGTGA | TGCTGGTGGTGCCTGAT |
| Rp49 | CGTAAACCGAAGGGAATTGA | GAAGAAACTGCATGGGCAAT |
