# Supplementary material for: Aggregation pheromone 4-vinylanisole promotes the synchrony of sexual maturation in female locusts
Source: eLife. 2022 Mar 8;11:e74581. doi: 10.7554/eLife.74581 (PMC8903828; doi:10.7554/eLife.74581)
Supplement: Transparent reporting form [file elife-74581-transrepform1.docx]

***eLife’s* transparent reporting form**

We encourage authors to provide detailed information *within their submission* to facilitate the interpretation and replication of experiments. Authors can upload supporting documentation to indicate the use of appropriate reporting guidelines for health-related research (see [EQUATOR Network](http://www.equator-network.org/%20)), life science research (see the [BioSharing Information Resource](https://biosharing.org/)), or the [ARRIVE guidelines](http://www.plosbiology.org/article/info:doi/10.1371/journal.pbio.1000412) for reporting work involving animal research. Where applicable, authors should refer to any relevant reporting standards documents in this form.

If you have any questions, please consult our Journal Policies and/or contact us: [editorial@elifesciences.org](mailto:editorial@elifesciences.org).

**Sample-size estimation**

- You should state whether an appropriate sample size was computed when the study was being designed
- You should state the statistical method of sample size computation and any required assumptions
- If no explicit power analysis was used, you should describe how you decided what sample (replicate) size (number) to use

Please outline where this information can be found within the submission (e.g., sections or figure legends), or explain why this information doesn’t apply to your submission:

- In this study, the sample size is determined according to previous publications in the locust species (Yang et al., 2014, PLoS Genetics, 10:e1004206; He et al., 2016, PNAS, 113(3):584-9; Hou et al., 2017, eLife, 6:e22526).
- The distribution of the first oviposition time and the consistency of sexual maturation (represented by the length of terminal oocytes) were analyzed using Levene’s test according to previous studies (He et al., 2016, PNAS, 113(3):584-9); Rohner et al., 2013, Science). The mean value of the first oviposition time between two groups was analyzed using Student’s t-test. One-way ANOVA followed by Tukey’s post-hoc test was used for multi-group comparisons. All experiments were performed with at least three independent biological replicates.
- Detailed information of sample size and biological replicate number for each experiment can be found in the figures.

**Replicates**

- You should report how often each experiment was performed
- You should include a definition of biological versus technical replication
- The data obtained should be provided and sufficient information should be provided to indicate the number of independent biological and/or technical replicates
- If you encountered any outliers, you should describe how these were handled
- Criteria for exclusion/inclusion of data should be clearly stated
- High-throughput sequence data should be uploaded before submission, with a private link for reviewers provided (these are available from both GEO and ArrayExpress)

Please outline where this information can be found within the submission (e.g., sections or figure legends), or explain why this information doesn’t apply to your submission:

- All experiments were performed for at least 3 independent biological replicates.
- The number of biological replications for each experiment is shown in the figures.
- We exclude the data that do not meet normal distribution for the analysis of sexual maturity, mRNA and protein levels, as well as JH titer measurement (Method Parts-Statistical analyses).
- The RNA-seq data reported in this study have been deposited in the Genome Sequence Archive (Genomics, Proteomics & Bioinformatics 2017) in National Genomics Data Center (Nucleic Acids Res 2020), Beijing Institute of Genomics (China National Center for Bioinformation), Chinese Academy of Sciences, under accession number CRA003038 that are publicly accessible at <https://bigd.big.ac.cn/gsa>

**Statistical reporting**

- Statistical analysis methods should be described and justified
- Raw data should be presented in figures whenever informative to do so (typically when N per group is less than 10)
- For each experiment, you should identify the statistical tests used, exact values of N, definitions of center, methods of multiple test correction, and dispersion and precision measures (e.g., mean, median, SD, SEM, confidence intervals; and, for the major substantive results, a measure of effect size (e.g., Pearson's r, Cohen's d)
- Report exact p-values wherever possible alongside the summary statistics and 95% confidence intervals. These should be reported for all key questions and not only when the p-value is less than 0.05.

Please outline where this information can be found within the submission (e.g., sections or figure legends), or explain why this information doesn’t apply to your submission:

- We have described the statistical analysis methods in detail in the Materials and Methods part-Statistical analyses.
- We have provided the raw data for figures with N < 10 as source data in the source data files.
- Detailed information of statistical tests is labeled in the figure legends. In addition, exact p-values and N are also shown in the figures.

(For large datasets, or papers with a very large number of statistical tests, you may upload a single table file with tests, Ns, etc., with reference to sections in the manuscript.)

**Group allocation**

- Indicate how samples were allocated into experimental groups (in the case of clinical studies, please specify allocation to treatment method); if randomization was used, please also state if restricted randomization was applied
- Indicate if masking was used during group allocation, data collection and/or data analysis

Please outline where this information can be found within the submission (e.g., sections or figure legends), or explain why this information doesn’t apply to your submission:

The samples were allocated into experimental group randomly, and no restricted randomization was applied (Methods part-Statistical analyses).

No masking was used during group allocation, data collection and data analysis.

**Additional data files (“source data”)**

- We encourage you to upload relevant additional data files, such as numerical data that are represented as a graph in a figure, or as a summary table
- Where provided, these should be in the most useful format, and they can be uploaded as “Source data” files linked to a main figure or table
- Include model definition files including the full list of parameters used
- Include code used for data analysis (e.g., R, MatLab)
- Avoid stating that data files are “available upon request”

Please indicate the figures or tables for which source data files have been provided:

Figure 1-source data: Raw data for first oviposition time and sexual maturity.

Figure 2-source data: Raw data for volatile contents and sexual maturity.

Figure 3-source data: Raw data for sexual maturity, JH titer, gene expression, and protein level.

Figure 4-source data: Raw data for JH titer, gene expression, and protein level.
